# Exploring the working conditions of disabled employees: a scoping review

**DOI:** 10.1186/s12995-023-00397-z

**Published:** 2024-01-30

**Authors:** Sophie Teborg, Lena Hünefeld, Tomke S. Gerdes

**Affiliations:** 1 Federal Institute for Occupational Safety and Health, Unit 1.2 Monitoring Working Conditions, Dortmund, Germany; 2 Department of Rehabilitation Sciences, TU Dortmund University, Dortmund, Germany

**Keywords:** Disability, Disabled Employees, Employment, Work, Working Conditions, Scoping Review

## Abstract

**Purpose:**

Disabled people are often overlooked in considerations about work design, which contributes to their exclusion from the labor market. This issue also reflects within research, as the body of knowledge on the working conditions of disabled employees remains relatively limited.

**Methods:**

A scoping review was conducted to assess the research landscape concerning the working conditions of disabled employees. Five databases have been searched, focusing on relevant studies published between 2017 and 2022.

**Results:**

One hundred fourteen studies were included in the review. It was found that social aspects of work appeared within a substantial portion of the examined studies. Furthermore, it became evident that the interplay of accessibility and flexibility provides an important dynamic to make work design both inclusive and feasible.

**Conclusion:**

The recurrent prominence of social aspects, accessibility, and flexibility across the studies shows common challenges and potentials within the work situation of disabled employees. This suggests avenues for future research and inclusive work design.

**Supplementary Information:**

The online version contains supplementary material available at 10.1186/s12995-023-00397-z.

## Introduction

When a person's specific health needs meet unfortunate environments, opportunities for participation quickly come to their limits. Consequently, individuals with health impairments find themselves disabled by various environmental barriers. The work environment is no exception to this.

Disabled people encounter a wide variety of challenges in their workplace that make work difficult or impossible. These can involve negative employer attitudes, inaccessible workplace environments, or problems in receiving work accommodations [1–3]. As a result, the employment rates of disabled people are lower worldwide than those of non-disabled people [4, 5]. Exclusion from the labor market not only fosters economic disadvantages and a higher risk of poverty for disabled people [6] but is also related to a sense of societal exclusion [4].

From an economic vantage point, the exclusion of disabled people in the labor market also engenders a disadvantage. Within a group comprising approximately 15% of the global population, there is a significant reservoir of labor potential [7]. Particularly in times of an exacerbating skills shortage, it should be attractive for employers to capitalize on this potential.

Nonetheless, the primary impetus for enhancing labor market inclusion should lie in the interests of those affected. Political demands for inclusion and participation manifest this. As evidenced by the Convention on the Rights of Persons with Disabilities (CRPD), 188 nations have pledged their commitment to advancing equitable rights for disabled people across all areas of life [8]. Article 27 of this Convention addresses the entitlement to “gain a living by work freely chosen or accepted in a labour market and work environment that is open, inclusive and accessible to persons with disabilities” [9]. Furthermore, the Convention enshrines the right of disabled people to protection against discrimination within the work context and to fair and favorable working conditions (Art. 27, 1).

Favorable working conditions for disabled employees enable them to effectively manage their health impairment and occupational responsibilities. In pursuit of this goal, the deployment of work accommodations has been found to be particularly useful [1, 10]. Such accommodations include flexible scheduling, modified job duties, and adapted work environments [1, 10]. However, research findings indicate that disabled employees often hesitate to request accommodations due to apprehensions about potential harm to their professional image or concerns about a lack of compliance by their employer [11]. This underscores another essential facet in this context: the treatment of disabled employees within the workplace. Indeed, authentic inclusion is not achieved until disabled employees experience a genuine sense of belonging to their work group and being valued for their uniqueness [12]. Relevant players in achieving authentic workplace inclusion comprise not only the immediate work teams of disabled employees but also leadership figures and the organization as a whole [13]. 

As previously stated, the CRPD constitutes the right of disabled people to fair and favorable working conditions. In addition, participating nations commit to the compilation of research data to monitor the execution of the Convention (Art. 31). The current state of research on the working conditions of disabled employees, however, faces criticism for its limited depth and high fragmentation.

First, insights apart from employment rates or income levels of disabled people are scarce [3, 14]. Furthermore, the research landscape is characterized as “a patchwork of findings on the experiences of people with very different conditions in varying and changing work contexts” ([15], p.4). The substantial fragmentation within the state of research is also mirrored in existing reviews, which tend to be focused on either a specific target group (e.g., autistic employees [16]) or particular work-related aspects (e.g., the role of the employer [17] or workplace accommodations [18]). However, comprehensive reviews integrating findings on the working conditions of disabled employees within a broader framework are currently absent.

Considering the lack of overarching findings in this field, this review was undertaken to present an outline of the prevailing working conditions of disabled employees as evidenced in the current research literature. The review addresses the following questions: “Which working conditions are present in the current research literature concerning disabled employees? What are opportunities and challenges for disabled employees in connection with these working conditions and overall work design?”.

To address these questions, a scoping review methodology was chosen. Scoping reviews are particularly suitable to map the literature on evolving topics because they place lower demands on the design of studies than systematic reviews [19] and thus allow a deeper insight into the literature. At the same time, scoping reviews claim certain quality standards that ensure their value for the research landscape.

This review aims to counteract the existing fragmentation within the research landscape and develop a holistic viewpoint of the working conditions of disabled people. Therefore, it focuses on in-company working conditions and the associated challenges and potentials. Even if, of course, the organization of work is dependent on the existing societal conditions and the respective national legal regulations. However, an advantage of the chosen approach is that it provides cross-national insight into working conditions.

To achieve this, the review systematizes the included studies regarding different levels of work (i.e., organizational-, team-, individual-level), presenting the findings in a novel yet comprehensive way. By shifting the focus away from specific groups or work issues and towards the working conditions of disabled employees as a whole, it becomes possible to unveil shared challenges and potentials at different levels and detect interdependencies. The lack of specification on disability types is aligned with the notion of designing work in a way that favors as many people as possible, elevating the chances of disabled people succeeding in the labor market. Proactively establishing favorable and inclusive working conditions for disabled employees is pivotal in cultivating inclusiveness in the working world. It introduces another perspective for inclusion within the employment context, complementing the usual reactive measures of individually tailored work accommodations.

## Materials and methods

The scoping review is led by the methodological framework of Arksey and O’Malley [20]. For reporting the review, the PRISMA Guiding Principles for Scoping Reviews (PRISMA-ScR) [21] are followed (see Additional file 1).

Considering library recommendations and initial keyword searches, the search was conducted in PubMed, PsycArticles, PsycInfo, PSYNDEX, and Embase for relevant articles published in English or German between 2017 and 2022. Furthermore, reference lists of existing reviews were manually searched. The search strategy was developed in an iterative process, whereby the initial search string was subsequently modified after evaluating the content and number of retrieved results. A combination of controlled vocabularies, such as Medical Subject Headings (MeSH), and selected keywords produced the most fitting results. The strategy was adjusted for each database to suit different search algorithms and supplemented with available search functions (i.e., proximity operators, “explode” function). The final search strategy embraced three overarching domains: the work context, the concerned population, and working conditions (see Additional file 2).

Guided by Polanin et al. [22], the research team developed a screening tool with inclusion criteria for the studies (see Additional file 3). To be included, the document in question had to 1) be a quantitative or qualitative study, 2) include a sample or subsample of disabled employees or third parties that give information about the work situation of disabled employees (i.e., employers, diversity managers), 3) have some information on the work situation beyond employment status or income, 4) take place in competitive employment, 5) not investigate transition processes or interventions and 6) involve information stemming from real-life situations, excluding experimental and vignette studies. The screening tool was used for decisions about inclusion throughout the whole review process.

For defining disability, this review follows a relational understanding, acknowledging that disability is constituted by the interaction of a health impairment with contextual factors, which subsequently impacts daily activities and participation [23]. While this is a widely used definition in modern disability research, the increase of aspects that are thought to connect to the concept of disability in the work context poses a challenge. As Lederer et al. [24] describe, it becomes increasingly difficult to determine whether the dimensions added to the concept of work disability over time are determinants, outcomes, or consequences of disability. This also reflects in existing studies, as they use various approaches to conceptualize and operationalize disabilities. The main reason for this is the high interdisciplinarity of the research field, resulting in different perspectives and research questions. Thus, narrowing the review to a specific definition of disability would also mean to narrow it to specific perspectives and fields of interest. As its exploratory nature is one of the things that characterize this review, a broad disability definition was chosen instead.

Consequently, disability is defined as an "umbrella term for impairments, activity limitations, and participation restrictions" ([23], p. 221). Thus, the baseline criteria for study populations to be included was that they either had a disability or a long-term health impairment. This approach allows the review to move at the interface of health impairments and (potentially) disabling working conditions. Therefore, it cannot only be examined how disabled employees deal with work but also what constitutes disability at work in the first place.

Using the final version of the screening tool, two reviewers screened a random sample of 100 titles and abstracts. After reaching a Cohen’s kappa [25] of κ = 0.89 for decisions about inclusion, the screening tool was found reliable for independent screening of remaining titles and abstracts. Nonetheless, the two reviewers were under regular exchange to resolve insecurities or upcoming questions. As the established inclusion criteria remained consistent, it was also agreed to use the tool for full-text screening. The first author conducted the full-text appraisal and consulted the research team in case of emerging questions.

For data extraction, the research team developed a data chart tailored to the objectives of this review. Ultimately, the data chart involved categories usually captured in reviews (i.e., year, country) as well as specific categories concerning disability and work. The first author collected the following study characteristics in data extraction: author(s), year of publication, country, study design and methods, study population, sample size, type of disability, work situation, and working conditions.

After data extraction, a thematic analysis according to Braun and Clarke [26] was performed. In the initial phase, the first author performed inductive data coding to generate different categories of working conditions. The categories were refined in an iterative process in consultation with the other authors. In further analysis, a deductive approach was used by assigning the generated categories to different levels of work.

The levels are inspired by Knight and Parker [27], who examined existing work design theories and found that these either focus on the organizational context, social systems or the individual work activity. Based on this, they summarized existing theories in three categories: *Organizational-System Approaches, Team Work Design Theories* and *Individual Work Design Theories.* Following this logic, the organizational level focuses on theories about work systems and human resource strategies, the team level on social systems and management in teams, and the individual level on specific job characteristics.

This threefold division of work allows to consider not only working conditions that are directly connected to the work activity itself but also the broader context determined by the social environment and the organization. Since these aspects are important for the work situation of disabled employees and authentic inclusion at the workplace, the approach has been found to provide a fitting framework for this review.

## Findings

**Fig. 1:**
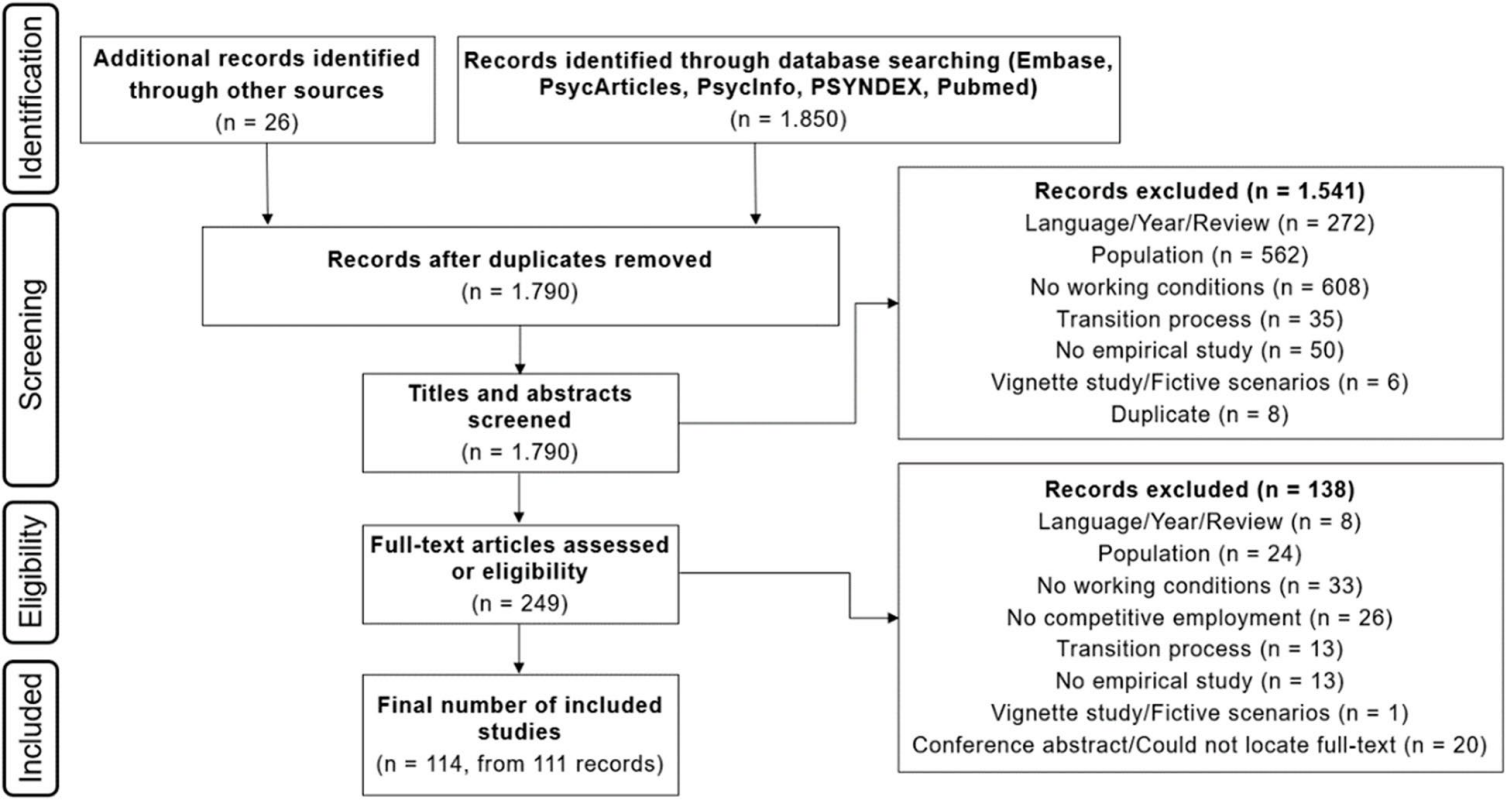
Flowchart of scoping review screening

Until 18th October 2022, the database search yielded 1.850 records (see Fig. 1). Twenty-six studies were identified through additional sources. After the removal of duplicates, there were 1.790 records left to screen. Applying the screening tool, 114 studies were identified as eligible for the review and thus built the final sample.

### Study characteristics

Most of the studies were conducted in the USA (*n* = 33), followed by the UK (*n* = 12), Canada (*n* = 11), the Netherlands (*n* = 10), and Germany (*n *= 9). Of the 114 studies, 89 used primary research data, 25 used secondary research data, and 1 used primary and secondary research data. Regarding study design, 62 studies used quantitative methods, 49 used qualitative methods, and 3 used a mixed-methods approach. Furthermore, far more studies were cross-sectional (*n* = 102) than longitudinal (*n* = 12). Slightly more than half of the studies involved a sample or subsample of employees with physical impairments (*n* = 62), followed by mental impairments (*n* = 45), cognitive impairments or divergencies (*n* = 35), sensory impairments (*n* = 32) and impairments related to activities of daily living without explicit specification of the type of health impairment (*n* = 10). It has to be noted that some the studies included more than one type of impairment in their sample, which is why the impairment types do not add up to 114. Additionally, 23 studies employed generic terms such as “disabled employees” or “chronic health conditions” without delineating classifiable health impairments or specific activity limitations.

### Working conditions of disabled employees

The assignment of categories to the three work levels yielded four categories on the organizational level, two categories on the team level and nine categories on the individual level (see Table 1). The organizational level includes information about organizational structures, the organizational culture and opportunities for advancement within an organization. The team level describes the direct social work environment, including supervisors and coworkers. At the individual level, categories are listed that determine the work activity itself, i.e., the contractual framework, work times, or psychological working conditions. The three levels of work and their subcategories are described in detail below. Thereby, the categories are illustrated by selected results of the included studies. This includes which working conditions of disabled employees have been taken into account in research so far, as well as the context in which they are discussed. This can mean whether specific working conditions are considered an opportunity or a challenge, whether they pose barriers, or how they can be accommodated.

**Table 1:** Categories of working conditions and studies assigned to each category

Categories	Relevant studies	n
***Organizational Level***		
**Organizational Structure**	[28–61]	**34**
Structures and processes	[28, 31–39, 42, 48, 50, 51, 56, 58]	16
Policies	[29, 32, 34, 40–42, 48, 50, 52, 56, 57, 60, 61]	13
Hierarchies and roles	[39, 43–48, 54, 59]	9
Awareness trainings	[34, 49, 53, 60]	4
**Organizational Culture**	[29, 31–36, 38–42, 47–53, 55–58, 60, 62–84]	**47**
Attitudes	[29, 31–34, 36, 38–42, 47–52, 55–58, 60, 62–69, 71, 72, 74, 76–79, 81, 82, 84]	40
Disability awareness and knowledge	[29, 34, 35, 39, 49, 52, 53, 64, 66, 69–71, 73, 75, 80, 83]	16
**Professional Growth**	[30, 35, 36, 38, 42–45, 47, 48, 50, 51, 54, 59, 60, 65, 69, 71–73, 75, 82, 85–96]	**34**
Careeer development	[30, 36, 38, 42, 43, 45, 50, 51, 54, 59, 60, 69, 71–73, 82, 85–87, 90–92, 94, 96]	24
Occupational trainings	[36, 38, 43, 44, 47, 48, 51, 54, 59, 65, 69, 75, 88, 93, 95, 96]	16
**Health Promotion**	[37, 38, 40, 47, 52, 55, 62, 65, 71, 72, 97, 98]	**12**
Occupational physicians	[37, 40, 52, 55, 65, 98]	6
Health programs	[38, 47, 62, 71, 72, 97]	6
***Team Level***		
**Leadership Quality**	[3, 31–33, 35–40, 42–44, 46, 48, 52, 55–58, 60, 62, 64, 65, 68, 69, 72, 73, 76, 78, 79, 81, 85, 89–91, 98–111]	**50**
Support	[31, 33, 36, 37, 39, 40, 55–57, 60, 62, 64, 72, 73, 76, 78, 79, 85, 89, 90, 99, 100, 106, 108, 111]	25
Attitudes	[3, 31, 36, 48, 57, 58, 62, 68, 72, 73, 79, 81, 85, 90, 91, 98–101, 103, 107, 110]	22
Relationships	[38–40, 44, 46, 57, 72, 73, 91, 104]	10
Disability awareness and knowledge	[35, 46, 52, 56, 57, 65, 72, 90, 103]	9
Communication and feedback	[43, 44, 48, 62, 64, 89, 90, 100, 105]	9
Control	[32, 42, 44, 58, 85, 104, 105]	7
**Team Climate**	[3, 28, 31–36, 38–60, 62–65, 68, 70–73, 76–83, 85, 89–91, 93–96, 98–102, 104–126]	**84**
Attitudes	[3, 31, 32, 35, 36, 38, 39, 41–48, 50, 52, 53, 55, 58, 62, 63, 65, 68, 71–73, 77–79, 81–83, 91, 93–95, 98–100, 102, 104, 105, 107, 109, 110, 113, 115, 118, 119, 122, 124–126]	54
Support	[3, 28, 33, 38, 41, 43, 54–57, 59, 60, 63, 64, 68, 72, 76–80, 85, 90, 93, 94, 96, 100, 106, 108, 111, 112, 116, 117, 120, 121, 123]	36
Relationships	[32, 34, 36, 40, 41, 53, 58, 70, 72, 73, 89, 99, 104, 114, 118]	15
Communication	[32, 34, 35, 43, 44, 49, 51, 58, 73, 90, 93, 96, 105]	13
Disability awareness and knowledge	[49, 65, 72, 101, 112]	5
***Individual Level***		
**Regulatory framework**	[1, 3, 28, 29, 32–34, 36–38, 41, 43–47, 49–51, 55, 57, 60, 61, 63–65, 67, 69–75, 77–79, 81, 82, 84, 86, 87, 89, 93–95, 97, 99, 100, 103–108, 110, 113, 114, 117, 123–137]	**74**
Contract	[1, 28, 29, 32, 34, 36, 37, 41, 43–47, 49, 55, 57, 61, 63–65, 67, 69–74, 77–79, 81, 82, 84, 86, 89, 93, 95, 97, 99, 103, 104, 106–108, 114, 117, 125–136]	58
Remuneration	[3, 38, 43, 45–47, 49–51, 60, 63, 64, 72, 75, 79, 82, 84, 87, 93–95, 99, 103, 105, 107, 110, 113, 123, 124, 129–131, 133, 137]	34
Leave Regulations	[32, 33, 43, 67, 72, 100]	6
**Work Time**	[1, 3, 31–33, 35, 37–40, 43, 48, 50, 51, 53–55, 57–60, 63–65, 67, 68, 71–73, 77, 81, 82, 84, 86, 88, 89, 94, 96, 98–101, 104, 105, 107, 108, 120, 125, 127, 132, 134, 138]	**52**
Flexibility	[1, 3, 31–33, 35, 37–40, 43, 48, 50, 53–55, 57, 60, 63, 67, 71–73, 77, 88, 94, 98–100, 104, 105, 127]	32
Breaks	[3, 33, 37, 43, 60, 72, 73, 81, 82, 89, 99, 105, 134]	13
Daily working hours	[51, 55, 58, 68, 94, 99, 108, 125]	8
Irregular work times	[55, 84, 86, 89, 94, 99, 132, 138]	8
Work-life balance	[37, 58, 65, 84, 96, 107, 120]	7
**Work Location**	[1, 3, 28, 30, 33, 34, 36, 38, 39, 42, 51, 53, 55, 56, 60, 63, 68, 69, 71, 72, 82, 83, 89, 94, 99, 100, 104, 105, 112, 114, 127, 139]	**32**
Working from home	[33, 34, 36, 39, 53, 55, 63, 68, 69, 71, 72, 94, 99, 100, 114, 139]	16
Commute	[1, 3, 28, 30, 33, 34, 38, 60, 72, 82, 89, 99, 104, 105, 127]	15
Changing work locations	[42, 51, 55, 56, 83, 99, 100, 112]	8
**Workplace**	[1, 3, 33–38, 40, 41, 43, 48, 50, 51, 54, 55, 58, 60, 62, 63, 68, 73, 75, 77, 80–83, 99, 100, 104, 105, 112, 117, 120, 121, 125, 134]	**38**
Physical environment	[3, 33, 34, 37, 38, 43, 50, 51, 54, 55, 63, 73, 75, 77, 80, 82, 99, 104, 105, 117, 120, 121]	22
Sensory environment	[35, 40, 41, 43, 48, 58, 60, 73, 83, 105, 112, 125, 134]	13
Spatial environment	[1, 37, 40, 58, 60, 68, 73, 80, 81, 105]	10
**Technology and Work Equipment**	[3, 30, 33, 34, 36, 37, 39, 40, 42–45, 53, 54, 58–60, 63, 64, 68, 69, 73, 77, 80, 82, 90, 91, 99, 100, 103–105, 112, 127, 134, 136]	**36**
Adapted and assistive technologies	[3, 36, 37, 40, 42, 43, 58, 63, 68, 80, 90, 91, 104, 112]	14
Digital technologies	[30, 34, 44, 45, 53, 82, 99, 103, 112, 134, 136]	11
Technology supply and support	[34, 40, 42, 69, 80, 91, 104, 112]	8
Work equipment	[33, 39, 54, 59, 73, 77, 100, 103]	8
Adapted and assistive equipment	[60, 64, 77, 105]	4
**Psychological Working Conditions**	[28, 33, 35–38, 40–43, 45, 46, 51, 53–55, 58, 59, 62–65, 72, 73, 76, 78, 82, 84, 88, 90, 92–96, 99, 100, 103–105, 107–109, 111, 116, 120, 128–131, 134, 135]	**52**
Work intensity	[28, 35, 36, 38, 40–42, 51, 53, 55, 58, 63–65, 72, 73, 90, 92, 94, 100, 104, 109, 114, 120, 129, 134]	26
Autonomy and decision latitude	[28, 33, 37, 38, 54, 59, 62, 63, 72, 76, 78, 94, 99, 107, 108, 111, 116, 128–131]	21
Cognitive requirements	[36–38, 42, 43, 46, 54, 58, 59, 82, 84, 100, 105, 131]	14
Variability of work	[43, 46, 54, 55, 58, 59, 63, 64, 72, 99, 100, 105]	12
Responsibility	[37, 38, 40, 46, 51, 72, 93, 104, 109]	9
Job security	[58, 73, 94–96, 107, 116, 131]	8
Psychological job demands	[38, 54, 108, 111, 116, 128, 130, 135]	8
**Social Working Conditions**	[33, 35, 37, 40, 41, 43–45, 47, 51, 54, 58–60, 93, 95, 100, 105, 109, 122]	**20**
Third party contact	[33, 37, 41, 43–45, 47, 58, 59, 93, 100, 109, 122]	13
Social requirements	[35, 40, 43, 51, 54, 58–60, 95, 105, 109]	11
**Physical Working Conditions**	[33, 37, 51, 54, 55, 59, 67, 72, 84, 99, 100, 106, 108, 128, 130, 134, 135]	**17**
Physical job demands	[54, 55, 59, 67, 84, 106, 108, 128, 130, 135]	10
Lifting and carrying loads	[33, 67, 72, 134]	4
Mobility	[33, 51, 100]	3
Body postures	[37, 99, 134]	3
Repetitive motions	[99, 134]	2
**Work Accommodations and Supports**	[1, 3, 30, 31, 35, 37–41, 46, 55, 60, 62, 64, 67, 72, 77, 82, 89, 90, 98–100, 127, 132, 135]	**27**
Work accommodations	[1, 31, 35, 37–40, 46, 55, 62, 67, 72, 77, 82, 98–100, 127, 135]	19
Human support	[1, 3, 30, 38, 41, 60, 64, 77, 89, 90, 127, 132]	12

*Legend 1* *:* *Cursive: Levels of Work;* Bold: Categories of Working Conditions

#### Organizational level

Working conditions at the organizational level are divided into four categories: Organizational structure (*n* = 34), Organizational culture (*n* = 47), Professional growth (*n* = 34), and Health Promotion (*n* = 12).

#### Organizational structure

Sixteen studies involve information on structures and operational processes within organizations. Among these, some studies discuss organizational flexibility [28, 31–34]. Organizational flexibility is thought to be related to the preparedness of organizations to create flexible work [34] and whether organizations allow different work modes [35] or work adjustments [33]. This includes whether employers are prepared to build flexible jobs in terms of work load, work times or mode of payment [34] or if employers allow spontaneous changes, i.e. when it comes to working from home [35].

Another facet that is addressed is operational processes. Studies scrutinize the absence of formalized processes to support disabled employees [36] and the complex nature of these processes [37]. In the course of this, it is also addressed whether there are fixed contact persons for disabled employees in organizations whom they can consult [35, 38, 39].

Organizational policies are referenced in thirteen studies. Examples encompass inclusion policies [34, 40, 41], policies for remote work [34], and policies for workplace accommodations [42]. Hierarchies and roles are discussed in nine studies. As illustrated by one study, flat hierarchies are posited to enhance access to “key decision makers” [39] within organizations. Additionally, the studies underscore the importance of disabled employees comprehending their roles within the organizational framework. Therefore, clear and congruent job descriptions, aligned with the actual job and employer expectations, are addressed [43–47]. Finally, four studies mention awareness training as a potential avenue to sensitize organizational members to the subjects of disability and inclusion.

#### Organizational culture

Forty studies offer insights into organizational attitudes. These reflect the degree of inclusivity within the workplace climate, involving the extent of supportiveness and understanding organizations display toward disabled employees. Within the studies, an inclusive culture is delineated as one that fosters equitable opportunities for all employees, values their diversity [48, 49], and normalizes disability [62]. Furthermore, aspects of the organizational culture such as understanding [63–65], trust [34, 40], respect [32, 47], and no tolerance for discrimination [42] are mentioned. Conversely, some studies thematize cultural aspects with adverse implications for disabled employees. These encompass rigid employer attitudes [34] as well as instances of stigmatization [66], discrimination [67], and doubt and suspicion [50] coming from the organization.

Another thematic strand pertains to the willingness of organizations to support and accommodate disabled employees. Along this trajectory, the studies indicate that some employers dismiss accommodation requests by disabled employees [51] or do not provide accommodations because of a lack of understanding [31, 52, 68, 69]. This also connects to disability awareness and knowledge within organizations (*n* = 16). As indicated by the studies, representatives of organizations often appear to possess inadequate knowledge regarding the nature of impairments [53, 64, 70], the workplace impacts on impairments [71], and the possibilities of support and funding for disabled employees [34, 35, 39, 52].

#### Professional growth

Regarding career development (*n* = 24), the studies especially illustrate the equity of career prospects for disabled employees compared to their non-disabled counterparts [54, 62–64]. The findings reveal instances where disabled employees perceive discrimination concerning promotions and career advancement [50] and non-disabled coworkers with lesser experience and qualifications are promoted before them [85]. Furthermore, two studies describe that disabled employees perceive so-called “glass ceilings”, hindrances that impede their career advancement [45, 51]. Among the sixteen studies encompassing information about occupational training, two principal aspects emerge: The first aspect revolves around the provision of training opportunities by the employer [38, 43, 51, 54, 69, 88]. The second aspect pertains to the accessibility of training and training materials for disabled employees [36, 38, 44, 69].

#### Health promotion

When it comes to health promotion, the most frequent theme is the occupational physician (*n* = 6). Two studies underscore the difficulties encountered by disabled employees in effectively utilizing their services: occupational physicians might lack visibility in organizations, have little time for employees, or are generally hard to access [37, 52]. Furthermore, studies report that occupational physicians lack knowledge regarding chronic conditions [65] or give inappropriate advice to disabled employees [55]. In addition, six studies incorporate insights into healthcare initiatives within the workplace. Disabled employees and supervisors perceive healthcare services as an important aspect when it comes to disease management and psychological support [48, 61, 71]. However, another study found that disabled employees are less likely to participate in health programs than non-disabled employees due to accessibility issues [68].

#### Team level

At the team level, there are two thematic areas: *Leadership quality* (*n* = 50) and *Team climate* (*n* = 84).

#### Leadership quality

The most frequent theme concerning leadership quality is support (*n* = 25). Supervisors are described as an essential source of support [56] and can function as a key to employment success for disabled employees [64]. Most importantly, they play a crucial role as gatekeepers in facilitating work accommodations [57]. Within the studies, supportive supervisors are recognized for their contributions to mitigating work intensity [72, 89], reorganizing tasks [37, 40, 62], and adjusting worksites [40]. Furthermore, the studies show that supervisors can provide emotional support and motivate disabled employees [55, 64]. Conversely, unsupportive supervisors can create barriers for disabled employees by withholding or disallowing accommodations [40, 99]. Among the studies that explore supervisor attitudes (*n* = 22), most focus on negative attitudes. These encompass a lack of understanding toward disabled employees [31, 72, 73, 90, 100], a failure to take them and their concerns seriously [68, 85], and manifestations of intolerance [101], stigmatization [36, 50, 91], and mistreatment [36, 50, 58, 102]. Then, again, some studies reflect positive attitudes, such as in supervisors that provide acceptance, understanding, and equitable treatment for disabled employees [57, 103] and respect and value them [62]. Good interpersonal relationships with supervisors (n = 10) are portrayed as positively influencing workplace integration [72], job satisfaction and performance [104], and the overall work environment for disabled employees [73]. Another salient aspect of the studies is the awareness and knowledge of supervisors regarding disabilities (*n* = 9). This relates to their knowledge regarding the capabilities and limitations of disabled employees [46] and how to accommodate them adequately [90, 103]. Knowledge also includes awareness of organizational support mechanisms [35] and relevant laws and regulations [52]. Furthermore, nine studies hold information on communication and feedback by supervisors. This includes their availability to employees [48, 89] and the depth and clarity of the feedback they offer [43, 44, 62, 90]. Finally, seven studies thematize control through supervisors, frequently addressing micromanagement [32, 44, 58, 104, 105].

#### Team climate

The most frequent theme regarding the team climate is the attitude of coworkers (*n* = 54). Within the studies, negative coworker attitudes prevail. A common focus lies on misconceptions and stigmatization directed towards disabled employees [31, 35, 36, 47, 65, 71–73, 78, 91, 98, 100, 104, 105]. Moreover, the studies underscore instances of unjust treatment faced by disabled employees, ranging from insulting and bullying [36, 41, 44, 58, 62, 73, 82, 119] to incidents of harassment and violence [95, 115, 122, 124]. Additionally, some studies address the phenomenon of coworker jealousy when disabled employees are granted work accommodations [45, 52, 53]. As such, negative attitudes are described as posing barriers [3, 77] and work difficulties [125] for disabled employees. Conversely, the studies delineate instances of positive coworker attitudes, manifesting as understanding [41, 62, 98], appreciation and valuing [38, 46, 99], and respect [32, 42, 63, 99, 107, 113]. Support from coworkers (*n* = 36) is noted as contributing to a good and empowering social environment for disabled employees [63, 94], wherein they can receive help [56, 68, 112], feel secure [78, 90], and experience inclusion [57]. In this line, support is portrayed as a facilitator of work for disabled employees [33, 55]. Closely related to support are interpersonal relationships with coworkers (*n* = 15). According to the studies, positive interpersonal relationships are pivotal for disabled employees to experience a sense of integration within the workplace [72] and develop a feeling of belonging [99]. In this way, good relationships at the workplace positively impact their overall work situation [32, 64]. However, negative relationships can yield additional barriers [70] and act as sources of stress [58]. Notable communication risks include miscommunication and misinterpretation [35, 49, 51, 58, 73] and a lack of communication [43]. Finally, five studies highlight the issue of disability awareness and knowledge within teams. As elucidated by some studies, the lack of awareness among coworkers connects to prejudices, miscommunication, and discrimination [49, 65, 72]. To address this, two studies propose the education of coworkers about disabilities as a potential solution [72, 112].

#### Individual level

The individual level is represented by nine categories: Regulatory framework (*n* = 74), Work time (*n* = 52), Work location (*n* = 32), Workplace (*n* = 38), Technologies and work equipment (*n* = 36), Psychological working conditions (*n* = 52), Social working conditions (*n* = 20), Physical working conditions (*n* = 17), and Work accommodations and supports (*n* = 27).

#### Regulatory framework

The prevailing subtheme within the regulatory framework is the employment contract of disabled employees (*n* = 58). Most of the studies use information on employment contracts for sample descriptions. This can include weekly working hours (full or part-time) and time limits (permanent or fixed-term). Some studies also explore the flexibility afforded to disabled employees in altering their employment contracts, such as transitioning from full-time to part-time arrangements [34, 70, 72, 135]. This flexibility is deemed significant because it facilitates the management of health fluctuations. Details regarding the income of disabled employees are present in thirty-four studies. Like the contractual situation, income is usually used to describe the sample. However, some studies address the perception of income in the context of appropriateness and fairness [29, 42, 81, 131]. This incorporates whether employees suspect to receive lower compensation due to their disabilities [3, 36, 77, 85]. Concerning leave regulations (*n* = 6), the most commonly addressed subjects involve employer approval of sick leave and the increase and flexibility of sick days [32, 33, 43, 67, 100]. Benefits of enhanced sick leave flexibility are that it allows disabled employees to respond to early warning signs of dynamic diseases [32] or claim inpatient hospitalization [100] without fearing for their job.

#### Work time

Regarding work time, the main focus within the studies centers on flexibility (*n* = 32). Flexibility involves adjustments to the start and end times of a work day or the total number of hours worked on a day. The studies elaborate that flexible work time offers benefits such as facilitating medical appointments [32, 67] and dealing with health fluctuations [99, 104]. Consequently, work time flexibility is depicted as a valuable accommodation for disabled employees [32, 53, 77]. In contrast, some studies picture rigid working hours as a potential challenge [33, 98, 99]. The second most prominent topic of work times revolves around breaks (*n* = 13), wherein flexibility also receives significant attention [43, 72, 72, 73, 89, 89, 99]. The length and frequency of breaks are also thematized [3, 37, 73, 82, 105]. Another topic is aligning break activities with individual needs, i.e., movement [33, 81] or eating warm meals [134]. Eight studies involve aspects of daily working hours. This concerns the length of work days [51, 55, 58, 94, 125] and working overtime [68, 99, 108]. In this context, prolonged and additional hours are depicted as challenges for disabled employees [51, 55]. Beyond that, one study portrays how disabled employees constantly work more hours than their coworkers because it takes them longer to complete tasks [68]. Regarding irregular working hours (*n* = 8), the studies encompass subjects like shift work [55, 84, 86, 99, 132, 138], unsocial work hours [89, 94]. Two studies highlight the challenges of shift work for disabled employees, prompting discussions about accommodations such as splitting shifts or avoiding evening and night shifts [55, 138]. Finally, work-life balance is mentioned in seven studies. In this context, the emphasis is on work-life balance as a pivotal component of the well-being, work capability, and work engagement of disabled employees [37, 65].

#### Work location

Sixteen studies address the subject of working from home. Generally, working from home is portrayed as providing flexibility for disabled employees [39, 63, 71, 100]. It can be a solution when disabled employees grapple with concentration difficulties in the office [36, 68] or when the strain of commuting to the workplace is too high due to fluctuating symptoms or medication side effects [33, 63, 71]. In contrast, two studies highlight potential drawbacks of working from home, noting that disabled employees might use this opportunity to conceal existing problems [53] or end up in social isolation [34]. Another topic is the commute to work (*n* = 15). Some studies describe bridging the distance between home and the workplace as a substantial barrier for disabled employees [34, 63, 104]. Correspondingly, other studies underscore providing transportation assistance as a valuable accommodation for disabled employees [1, 3, 38, 60, 99, 127]. Considerations regarding changing work locations (*n* = 8) frequently revolve around the challenges of inadequate accessibility [51, 56, 83, 99]. On the other hand, changing work locations can facilitate disease management, as exemplified by an employee who viewed it positively that they could work during their routine medical appointments [55].

#### Workplace

Twenty-two studies offer insights into the physical work environment of disabled employees. The dominant focus lies on workplace accessibility, which entails the presence of elevators, ramps, and accessible toilet rooms [3, 50, 55, 63, 77, 104] as well as adaptions made for disabled employees in the physical environment [33, 37, 75, 80, 105]. Regarding the sensory environment (*n* = 13), frequent topics are noise levels [3, 41, 48, 58, 60, 73, 105, 112, 125, 134], lighting conditions [35, 41, 43, 73, 105, 125, 134], temperature [58, 105, 134], and air quality [58, 83, 125] in the workplace. Ten studies address the spatial situation, which involves whether disabled employees occupy single or shared offices [37, 58, 60, 68, 80, 81]. In this context, retreating to separate workspaces when needed is described as a possible accommodation for disabled employees [37, 40, 73].

#### Technology and equipment

The most mentioned topic in this category surrounds adapted and assistive technologies (n = 14). These could be adapted computers and smartphones [3, 104], special keyboards and mice [37, 80], microphone systems [40], captioned telephones [112], dictate and spellcheck software [68, 91], and digital reminders and notetaking applications [43]. Moreover, eleven studies include information on the general utilization of digital technologies in the workplace. This encompasses tools such as computers [53, 134, 136], telephones and e-mails [111], and chat programs [44]. While the potential of digital technologies to surmount barriers for disabled employees is underscored in some studies [30, 99], it is also acknowledged that they can create new barriers when lacking accessibility [34, 82]. The usability of digital and assistive technologies is also contingent on the technological support extended by the employer (*n* = 8). The studies reveal recurring issues such as inadequate IT support, delayed or absent updates, and compatibility conflicts between mainstream and assistive technologies [69, 80, 91, 104]. In addition to technology, eight studies consider work equipment’s availability and functionality. Additionally, four studies address specific equipment employees use due to their impairments, such as sensory aids [64].

#### Psychological working conditions

The predominant subject within psychological working conditions is work intensity (*n* = 26), delineated by workload and work pace. High work intensity is acknowledged as a challenge for disabled employees [51, 104], as it engenders difficulties for the work situation of disabled employees [73, 109, 120] and their mental health [36, 53, 58]. Feasible accommodations described in the studies include the reduction and differentiation of workloads [40, 53, 72], a slower work pace [55, 134], less stressful duties [55, 134], less pressure [63], and increased flexibility regarding deadlines [41]. Concurrently, disabled employees hesitate to voice concerns about high workloads [65] as they try to embody the ideal worker [42]. Next, twenty-one studies include aspects of autonomy, such as skills discretion, decision latitude, and job control. According to the studies, autonomy facilitates disease management [78] and allows disabled employees to succeed in the workplace [33, 37, 62, 99]. Consistent with this perspective, autonomy is associated with heightened work ability [76] and a decreased risk of leaving the workforce [111] among disabled employees. Conversely, study findings indicate that disabled employees experience less autonomy than their non-disabled counterparts [129, 130]. This is supported by disabled employees expressing the need for more autonomy at work [63, 72]. Cognitive requirements are thematized in fourteen studies. Being intellectually stimulated and able to use one’s competencies is underscored as pivotal for disabled employees to feel valued and cope with work [37, 46]. However, the studies reveal that disabled employees frequently report being engaged in basic or unskilled work or having too few cognitive requirements [42, 43, 46, 82, 104, 105]. Only in two studies disabled employees perceive cognitive demands as being too high [36, 58]. Regarding work structure (*n* = 12), disabled employees frequently express the need for consistent routines [55, 64, 105] and a structured job [63]. Similarly, the absence of established routines and the inability to anticipate future tasks are identified as sources of stress [58, 99]. Further challenges are lonely and monotonous tasks [46] and repetitive work [43]. Responsibility (*n* = 9) is frequently portrayed as a challenge for disabled employees, prompting discussions about reduced responsibility as an accommodation measure [37, 40, 72, 104]. On the other hand, responsibility can evoke a sense of being irreplaceable to disabled employees [46] and thus serve as a source of motivation and satisfaction [104]. Eight Studies include aspects of job security, wherein the expectation of job loss is cited to negatively impact work [73] and be a source of stress [58]. Finally, eight studies refer to psychological working conditions on a less detailed scale under the term “psychological job demands”.

#### Social working condition

Thirteen studies thematize contact with third parties such as customers or clients, making it the most common theme among social working conditions. Relatedly, the studies highlight challenges for disabled employees, such as handling customer communication [43, 44] and being mistreated through third parties [58, 122]. As a possible accommodation, the studies touch upon the possibility of reducing customer contact [37, 41]. Furthermore, eleven studies involve insights into the social demands of the job, including required contact with others at work [35, 43, 54, 59, 95, 109] and the need for participation in public events or conferences [58, 51]. Moreover, one study describes that the requirement for networking activities can pose a challenge for disabled employees in the case of communication difficulties [51].

#### Physical working conditions

Many studies refer to physical working conditions on a broader level, calling them physical job demands (*n* = 10). High physical demands are generally described as a risk factor for dropping out of work [55, 67], while lower physical demands positively predict working beyond retirement for disabled employees [84]. At the same time, one study shows disabled employees have greater physical demands than non-disabled employees [130]. On a more detailed level, specific aspects are mentioned, one of which is lifting and carrying loads (*n* = 4). Given the demanding nature of this task, a possible accommodation involves exempting disabled employees from the duty to lift or carry heavy loads [72]. Mobility (*n* = 3) constitutes another facet, including the requirement to walk and be physically active at work. Furthermore, the studies mention different body postures (*n* = 3), considering aspects like the degree of postural variability [99] and whether body postures are painful or tiring [134]. Beyond that, two studies mention repetitive motions as a physical working condition.

#### Work accommodations and supports

In addition to possible work accommodations already discussed, nineteen studies reference accommodations on a deeper level, fundamentally changing the job itself or how work is done. These encompass scenarios where disabled employees are assigned fewer, less demanding, or different tasks [37, 72, 82, 98, 135] or share tasks with coworkers [39, 67]. One study also describes the flexible assignment of work tasks to fit employees’ needs and skills, a practice known as job carving [62].

Human support is another facet of overarching work accommodations (*n* = 12). On the one hand, human support can be provided through personal assistants [1, 67, 127], sign language interpreters [90, 127], or interpreters for people who are blind [127]. On the other hand, some studies elucidate how coworkers can offer formal support as well when there are arrangements for assistance [1, 30, 127].

## Discussion

Taking a comprehensive perspective on disabilities and work contexts, the review offers insight into a wide range of working conditions explored within studies focusing on disabled employees.

The substantial volume of identified studies underscores the notion that the scarcity of knowledge in this area is not a consequence of too little research; instead, it emanates from difficulties associated with synthesizing existing findings. As posited initially, the research landscape demonstrates a high degree of fragmentation attributable to the heterogeneity of disability types and work contexts under consideration. However, the extensive research framework employed in this review also showed that specific aspects of work were mentioned particularly frequently across the included studies, covering various disabilities and work contexts.

This was especially evident in working conditions entailing the social environment of disabled employees. Notably, team climate was the most frequent category among all. Additionally, the categories of leadership quality and organizational culture surfaced across numerous studies. On the one hand, this indicates that social aspects play an essential role in shaping the work situation of disabled employees. On the other hand, it accentuates the multifaceted nature of social inclusion in the workplace. This is in line with Shore et al. [13], who describe forms of inclusion such as workgroup inclusion, leader inclusion, and organizational inclusion. Likewise, themes pertinent to inclusion, such as acceptance, support, or stigmatization, appear in the review concerning the organization, supervisors, or coworkers. Existing research also emphasizes the interplay among these stakeholders. In their study, Glade et al. [140] illustrate the responsibilities of employers, supervisors, coworkers, and disabled employees in fostering inclusive work environments. By doing so, they underscore that inclusive practices should especially be initiated at higher organizational levels. In line with this, a review by Jansen et al. [17] shows that workplace participation of disabled employees can especially be facilitated by supervisors who provide work accommodations and are supportive. The high relevance of social aspects within the workplace may be partly attributed to its connection to other working conditions. As evidenced in several parts of the review, the provision and implementation of work accommodations are highly dependent upon organizational support, endorsements from leaders, and acceptance among peers.

Another important discovery lies in the recurrent mention of aspects regarding accessibility. The impact of accessibility can be seen at the organizational level in organizational structures, occupational training, and healthcare programs, and at the individual level in the workplace, in the use of digital technologies, and in different work locations. The thematic categories incorporating elements of accessibility found within the review align with what the CRPD emphasizes concerning accessibility. As stated by the CRPD [9], accessibility entails equal access “to the physical environment, to transportation, to information and communications, including information and communications technologies and systems, and to other facilities and services open or provided to the public” (Art. 9, 1). As the review showed, most studies explore accessibility through subjective appraisals of barriers experienced by disabled employees without using theoretical frameworks. This, however, limits the conclusions that can be drawn for work design to individual contexts. Notably absent are findings regarding accessibility through systematic theoretical frameworks. It would be worth considering whether accessibility could be investigated more specifically by orienting on established guidelines. The Web Content Accessibility Guidelines (WCAG) 2.0 [141] furnish a conceptual underpinning for digital accessibility, predicated upon four fundamental principles: Web content has to be perceivable, operable, understandable, and robust. An interesting avenue for exploration could be how these principles can be used to scientifically assess technological equipment in the workplace. Furthermore, it could be useful to investigate whether these principles can be translated to other work areas.

An alternative approach for assessing accessibility within the work context is following the principle of universal design. In universal design, products or environments are conceived for usability for all individuals [142]. By integrating accessibility from the outset, the universal design approach obviates the necessity for adaptations or specialized functionalities for distinct users. In their publication, Sheppard-Jones et al. [143] expound upon the advantages disabled employees experience by implementing universal design in the workplace. They argue that the elevated level of accessibility inherent in universal design negates the need for individual work accommodations. Consequently, the risk of non-disabled coworkers perceiving preferential treatment of disabled employees by the employer diminishes, thereby potentially fostering positive implications for issues such as stigmatization or discrimination.

However, given the heterogeneous requirements for accessible working conditions that come with different types of health impairments, it becomes evident that the goal of attaining universal accessibility might be elusive in certain instances. This leads to another crucial aspect frequently emphasized in the studies: flexibility.

Flexibility describes the possibility of making changes and adjustments to working conditions. As becomes evident in the studies, a certain extent of flexibility at work proves essential for cultivating favorable working conditions for disabled employees. Thereby, flexibility appears in multiple forms. It can mean being able to work from home, to work more or fewer hours a day, or to reduce demands such as workload, physically strenuous work, or customer interactions. Aspects of flexibility were thus found in various categories, including the regulatory framework of a job, work times and locations, and psychological, physical, and social working conditions. Next to this, flexibility also appeared on the organizational level. Thereby, organizational flexibility describes the amount of flexibility employers allow in work design, making it an important prerequisite for flexible working conditions.

After focusing on the positive aspects of flexibility, it has to be considered that flexibility can also be perceived as a demand by disabled employees, as in the case of changing work locations or lack of work routines. However, the majority of the studies portray flexibility as a facilitator for the management of health impairments in occupational settings. Therefore, the exploration of flexible work design emerges as a promising avenue for setting new perspectives on the work situation of disabled people. One way to do this involves focusing on customized employment, a form of work design that uses several strategies to find employment solutions benefitting both the employer and the employee. The strategies encompass the selection of specific tasks from an existing job (job carving), the compilation of tasks from several jobs (job negotiation), the creation of novel jobs (job creation), the distribution of a job across multiple employees (job sharing), and the facilitation of self-employment opportunities [144]. Although existing findings suggest that customized employment enhances the quality of employment experienced by disabled people, the evidence regarding these practices remains limited due to a lack of randomized controlled trials [145, 146].

Apart from institutionalized measures such as customized employment, flexibility in work design can also manifest in more subtle forms. An example of this is idiosyncratic deals (i-deals) for disabled employees. I-deals are nonstandard work arrangements resulting from individual negotiations between employees and organizational stakeholders such as supervisors or human resource managers [147]. A notable advantage of i-deals is their empowerment of employees to customize their job proactively. Nevertheless, study findings indicate that i-deals are linked to certain preconditions, including organizational flexibility, an “ability” mindset on the employer’s part, and effective negotiation strategies [148]. Therefore, current research also investigates how representatives can support disabled employees in negotiating i-deals (ibid.).

Building on the finding that the principles of accessibility and flexibility constitute two fundamental components for favorable working conditions of disabled employees, it could be useful to consider how these principles might interact in work design. According to Sträter [149], work design should integrate the principles of homogeneity and flexibility equally. In advocating for such an approach, he accentuates the need for compromise: recognizing that work design can never be universally congruent for all employees, he advocates for cultivating the greatest possible scope for application within the broadest possible spectrum of limitations.

On a further note, it cannot be emphasized enough that the extent to which the principles of accessibility and flexibility are integrated into working conditions depends on the people who design them. In this line, it can be assumed that an accepting and supportive social environment promotes accessibility and flexibility. This becomes all the more relevant considering that neoliberal labor markets in most Western industrialized countries tend to strain social relationships by creating competition and economic pressure in many workplaces [150]. Furthermore, neoliberalism measures a person’s worth exclusively on their productivity and the subsequent profit they may generate. These characteristics of today’s labor markets may partly explain the frequent mention of negative attitudes, stigmatization, and discrimination toward disabled employees in the studies. Therefore, future research should examine these attitudes and investigate how organizations can stop stigmatization and discrimination and foster a climate of appreciation and support towards disabled people. In the same way, the results should also be examined against the background of different national labor markets and regulations to work out the interplay between in-company working conditions and societal orientation. Accordingly, not only work itself but also the structures in society and the labor market must be transformed in a way that promotes inclusion.

Another important finding is that aspects of the social environment, accessibility, and flexibility are relevant across all levels of work. Consequently, it is crucial to investigate the interplay of working conditions at the organizational, team, and individual levels. This is also suggested in existing research [151]. At the same time, it needs to be remembered that this is an idealized view and that, in reality, there is a high overlap between levels. In order to cope with this complexity, studies that can provide a comprehensive view of work situations are needed. For example, it could be useful to consider case studies in organizations that examine inclusive work design.

Lastly, it has to be acknowledged that the way in which disabled employees are affected by working conditions also depends on different types of disabilities. Nevertheless, the question remains whether the typification of disabilities based on specific health impairments is always useful, as the results and subsequent recommendations in such studies are usually very specific. One consideration would be to form samples according to other criteria and thus create greater relevance for practical work design. For example, more attention could be paid to the *interaction* between health impairments and the environment, i.e. by focusing on activity restrictions such as mobility or communication issues in work settings. At the same time, this could shift the focus away from the individual impairments, which always harbors the risk of overlooking environmental barriers.

### Limitations

Some limitations warrant consideration when interpreting the review’s findings. First, the search scope was confined to five databases, raising the concern that pertinent studies remained undetected. Furthermore, the studies were not subjected to quality assessment, impeding statements about the relevance of individual study findings. While Arksey & O’Malley [20] explicitly state that quality assessment is not a part of their scoping review framework, subsequent literature discusses quality assessment as a helpful tool within scoping reviews to evaluate the state of research [19]. Another constraint entails the variety of disability definitions. Although an expansive disability conception enabled the review to adopt a broad perspective, this approach amplifies the potential for overgeneralization [15, 152]. This applies both to different definitions and to types of disabilities. However, the myriad of disability definitions constitutes a known methodological challenge in disability research [153]. Therefore, it is proposed to choose the most suitable disability definition based on the intended research purpose [154]. Another limitation is the lack of consideration of different national contexts, given that legislation on work and disability diverges considerably across countries. However, the exclusive focus on OECD nations is assumed to mitigate this issue. Finally, classifying working conditions into different work levels merely reflects an idealized view. In reality, working conditions and work levels cannot be so clearly distinguished. Therefore, it needs to be taken into account that the proposed structure of three work levels and the subsequent assignment of working conditions to these levels may influence the interpretation of results.

### Conclusion and recommendations

To ensure the participation of disabled people in the labor market, it is imperative to design favorable and inclusive working conditions for them. This endeavor, however, necessitates a foundation of sound scientific knowledge.

The present review has shown the diverse approaches taken in researching the work situation of disabled employees. However, certain commonalities became apparent across different studies. These shared elements present important potentials for fostering inclusive working conditions, encompassing aspects of the social environment, accessibility, and flexibility at work.

The provision of accessibility is especially relevant regarding structures and processes within the organization, provided training and health programs, the physical workplace environment, and available technologies and equipment. For example, employers can foster accessibility by designating fixed contact persons to support disabled employees and implementing processes for requesting workplace accommodations. Regarding flexibility, employers can deliberate whether they can offer flexible work arrangements in hindsight of work times and locations, possibilities of task reassignment, or other individual agreements. Lastly and arguably most crucially, employers must ensure an inclusive social environment within their organization. This encompasses the acceptance and appreciation of disabled employees and eliminating possible negative attitudes toward them displayed by leaders, coworkers, or other organizational representatives. Diversity statements that explicitly mention disabled people and oppose any form of discrimination against them can be helpful for this. Another possible measure is diversity or anti-discrimination training for staff, provided that existing structures allow learned knowledge and strategies to be applied.

To strengthen inclusion in the labor market as proposed by the CRPD, it is essential that these suggestions are not considered as additional measures when disabled employees are hired but understood as fundamental principles that guide future work design.

### Supplementary Information

**Additional file 1:** Preferred Reporting Items for Systematic reviews and Meta-Analyses extension for Scoping Reviews (PRISMA-ScR) Checklist.

**Additional file 2: Appendix 2. **Example of a complete search strategy for one database (PsycArticles).

**Additional file 3: Appendix 3. **Screening tool with inclusion criteria for relevant studies.

## Data Availability

The datasets generated and analyzed during the study are available from the corresponding author upon reasonable request.
