# Supplementary material for: Exploring the working conditions of disabled employees: a scoping review
Source: J Occup Med Toxicol. 2024 Jan 30;19:2. doi: 10.1186/s12995-023-00397-z (PMC10826256; doi:10.1186/s12995-023-00397-z)
Supplement: Supplementary file 2 — Additional file 2: Appendix 2. Example of a complete search strategy for one database (PsycArticles). [file 12995_2023_397_MOESM2_ESM.pdf]

Sophie Teborg, Lena Hünefeld, Tomke S. Gerdes
Journal of Occupational Rehabilitation
teborg.sophie@baua.bund.de

Appendix 2 – Example of a complete search strategy for one database (PsycArticles)

	Subject Headings	Additional keywords
Concept 1: Work	MM "Occupations" OR MM "Sheltered Workshops" OR MM "Supported Employment" OR MM "Employment Status" OR MM "Vocational Rehabilitation"	Labor OR Work* OR Employment OR Job* OR (Integration OR Inclusion) W1 (compan*) OR (Integrated OR Sheltered) W1 (Employment) OR "social firm*"
Concept 2: Disabilities	DE "Disabilities" OR DE "Disability Management" OR DE "Learning Disabilities" OR DE "Multiple Disabilities" OR DE "Reading Disabilities" OR MM "Special Needs" OR MM "Disabled Personnel" OR DE "Disorders" OR DE "Adventitious Disorders" OR DE "Atypical Disorders" OR DE "Behavior Disorders" OR DE "Chronic Illness" OR DE "Communication Disorders" OR DE "Congenital Disorders" OR DE "Disabilities" OR DE "Disorder Attributes" OR DE "Lesions" OR DE "Mental Disorders" OR DE "Physical Disorders" OR DE "Sensory Integration Dysfunction" OR DE "Treatment Resistant Disorders"	OR "disabled employee" OR "disabled employee" OR (Disab*) N2 (employee* OR employee* OR intellectual OR sensory OR psychological OR physical OR mental) OR (Impair*) N2 (employee* OR employee* OR intellectual OR sensory OR psychological OR physical OR mental) OR (Physical OR functional OR mobility) W1 (limitation)
Concept 3: Working conditions	DE "Working Conditions" OR DE "Job Enrichment" OR DE "Noise Levels (Work Areas)" OR DE "Occupational Safety" OR DE "Telecommuting" OR DE "Work Rest Cycles" OR DE "Work Week Length" OR DE "Workday Shifts" OR DE "Working Space" OR DE "Job Characteristics" OR DE "Job Demands" OR DE "Job Resources" OR DE "Job Security" OR DE "Work Load" OR DE "Work Scheduling" OR DE "Flextime" OR MM "Quality of Work Life" OR DE "Job Performance" OR DE "Employee Efficiency" OR DE "Employee Productivity" OR MM "Job Involvement" OR MM "Employment Discrimination" OR MM "Employee Engagement" OR MM "Occupational Mobility" OR DE "Career Development" OR DE "Career Change" OR DE "Personnel Placement" OR DE "Personnel Promotion" OR MM "Job Satisfaction" OR MM "Work-Life Balance" OR DE "Occupational Stress" OR DE "Compassion Fatigue" OR DE "Employee Characteristics" OR DE "Employee Absenteeism" OR DE "Employee Attitudes" OR DE "Employee Efficiency" OR DE "Employee Motivation" OR DE "Employee Productivity" OR DE "Employee Retention" OR DE "Employee Skills" OR DE "Employee Turnover" OR DE "Employee Well Being" OR DE "Job Experience Level" OR DE "Job Knowledge“)	(Job OR labor OR work*) N2 (condition* OR demand* OR resource* OR characteristic* OR situation* OR load OR environment OR performance OR productivity OR qualification* OR satisfaction OR motivation OR disclosure OR accommodation OR strain OR harassment OR identification OR conflict* OR abuse OR task*) OR (shift) N2 (work OR night) OR (social) N2 (support OR relationship* OR context) OR (training) N2 (opportunit*) OR "trade union*" OR overtime OR downsizing OR workplace OR collegiality OR "work life balance"
Exclusion criteria (“NOT”)	MM "Unemployment“ OR MM "Home Care" OR MM "Home Environment"	/
	AG adolescence OR AG childhood OR AG school age OR AG preschool age OR AG infancy OR AG neonatal OR AG aged OR AG very old	
