# Supplementary material for: Exploring the working conditions of disabled employees: a scoping review
Source: J Occup Med Toxicol. 2024 Jan 30;19:2. doi: 10.1186/s12995-023-00397-z (PMC10826256; doi:10.1186/s12995-023-00397-z)
Supplement: Supplementary file 3 — Additional file 3: Appendix 3. Screening tool with inclusion criteria for relevant studies. [file 12995_2023_397_MOESM3_ESM.pdf]

Sophie Teborg, Lena Hünefeld, Tomke S. Gerdes

teborg.sophie@baua.bund.de

Appendix 3 – Screening tool with inclusion criteria for relevant studies

General information

- Publication in 2017 or later
- German or English language
- No Reviews/Meta-Analysis
- No Editorial, Erratum, Book Review or similar

Sample

- Sample consists of disabled employees or people that give information about disabled employees (proxies, co-workers, managers etc.)

Contents/Research question

- Study examines working conditions
- No exclusive focus on employment status or income
- No exclusive focus on transformative processes (return to work, application procedure)

Study design

- Quantitative or qualitative study design
- No examination of fictive scenarios (i.e. vignettes)
